# New Taxon-Specific *Heterobasidion* PCR Primers Detect and Differentiate North American *Heterobasidion* spp. in Various Substrates and Led to the Discovery of *Heterobasidion irregulare* in British Columbia, Canada

**DOI:** 10.3390/pathogens8030156

**Published:** 2019-09-18

**Authors:** Simon Francis Shamoun, Craig Hammett, Grace Sumampong, Xiang Li, Matteo Garbelotto

**Affiliations:** 1 Pacific Forestry Centre, Canadian Forest Service, Natural Resources Canada, 506 West Burnside Road, Victoria, BC V8Z 1M5, Canada; Grace.Sumampong@Canada.Ca; 2 Department of Forest and Conservation Sciences, University of British Columbia, 2424 Main Mall, Vancouver BC V6T 1Z4, Canada; craigahammett@gmail.com; 3 Canadian Food Inspection Agency, 93 Mount Edward Road, Charlottetown, PEI C1A 5T1, Canada; Sean.Li3@Canada.Ca; 4 Department of Environmental Science, Policy and Management, University of California, 54 Mulford Hall, Berkeley, CA 94720, USA; matteog@berkeley.edu

**Keywords:** elongation factor 1 alpha, glyceraldehyde 3-phosphate dehydrogenase, *Heterobasidion* species, molecular identification, phytosanitary, wood products trade

## Abstract

*Heterobasidion annosum sensu lato* is a species complex of pathogenic white-rot wood decay fungi which cause root and butt rot in conifer and hardwood species across the Northern hemisphere. Annual losses to forest managers are valued in the billions of dollars, due to tree mortality, reduction in timber yield, and wood decay. In North America, *H. irregulare* and *H. occidentale* have a partially overlapping host and geographic range, cause similar disease symptoms and produce similar fruiting bodies, making discrimination between the two of them often difficult. We developed two sets of primers that bind specifically to conserved, but species-specific portions of glyceraldehyde 3-phosphate dehydrogenase and elongation factor 1α alleles. The method is sensitive enough to detect either species from infected wood. Analysis of North American isolates has further clarified the distribution of both species on this continent, including the detection of *H. irregulare* for the first time on ponderosa pine (*Pinus ponderosa*) and eastern white pine (*Pinus strobus*) in British Columbia. This method has the potential to be a valuable tool for the detection of the pathogen in exported/imported wood products, as well as for the further identification and assessment of the distribution of North American *Heterobasidion* species.

## 1. Introduction

*Heterobasidion annosum* (Fr.) Bref. sensu lato (s.l.) is a significant forest pathogen that causes root and butt rot primarily in conifers across the Northern Hemisphere. The tree disease caused by *Heterobasidion* spp. is referred to as Annosus root and butt rot, and the mortality it causes in old growth forests is regarded as an important driver of forest turnover and natural biodiversity. However, damages to managed forests and plantation forestry include wood quality loss, timber yield reduction and tree mortality, and have been valued up to 790 million annually in the EU alone [1], making it the most important disease of conifers worldwide. Globalization has resulted in human-induced long-distance movement of this pathogen [2], and climate change has increased disease risk in sites that historically have not been challenged by this pathogen [3].

The genus *Heterobasidion* has been recently split into three genetically distinct clusters (*H. annosum, H. insulare* and *H. araucariae*) containing a total of 12 species [4] and a newly described hybrid taxon [5]. The *H. annosum* cluster includes two North American partially intersterile species, namely *H. irregulare* and *H. occidentale* [6], and a newly described hybrid taxon genetically distinct from the other two [5] In Europe, three species are currently recognized within the *H. annosum* species complex, namely *H. parviporum*, *H. annosum* sensu stricto (s.s.) and *H. abietinum* [7]. The Asian *H. insulare* cluster includes six species, all believed to be mostly saprophytic [4], while the species *H. araucariae* appears to represent a third monophyletic cluster with a geographic range spanning over Australia, New Zealand and adjacent regions [8].

*Heterobasidion irregulare* is widespread across Eastern North America forests from Quebec south to Florida and Central Mexico, in US Midwestern forests, [9], and in Pacific forests from Washington State (USA) to Baja California [10]. Its primary hosts are pine (*Pinus*) species, although scented cedar (*Calocedrus*) and juniper (*Juniperus*) are also common hosts. It can also be locally abundant on Pacific madrones (*Arbutus menziesii*) and Manzanitas (*Arcostaphylos* spp.). Rare infections of Douglas-fir (*Pseudotsuga*), a known host of *H. occidentale,* have also been reported [6]. *Heterobasidion irregulare* was accidentally introduced into Italy, presumably on infected wood products, during WW2 [11]. It is currently infesting Mediterranean pine forests around Rome, where it has proven to be significantly more widespread than the native *H. annosum*, due to its increased sporulation rates, and higher wood decay saprobic ability [12,13].

*Heterobasidion occidentale* is located exclusively on the west coast of North America, from Alaska to southern Mexico, and as far east as Colorado [10]. It has a broader host range than *H. irregulare*, and it is a pathogen of primary concern for hemlock (*Tsuga*), and true-fir species. However, it has also been found on several other species, including western red cedar (*Thuja plicata*), the giant sequoia (*Sequoiadendron*), coast sequoia (*Sequoia*) [14], Douglas-fir, spruce, larch, and even broadleaf species, such as alder (*Alnus*). 

The morphology of the fruiting bodies of the two North American species is primarily driven by site ecology [6], and many traits are overlapping between the two, making their diagnosis based on morphology alone quite challenging. A preliminary differentiation may be done through host, symptoms, and location; however, true differentiation can only be achieved through inter-sterility mating or DNA sequencing, both of which are time-consuming, and may require culturing in vitro of the pathogen. Differentiation based on mating tests can also be confusing because intersterility between the two is only partial [10,15]. To further complicate matters, hybrids between the two North American species have been identified in California and Montana [16,17].

Molecular diagnostic methods, including conventional and real-time PCR approaches, have been a heavily researched topic for the potential identification and differentiation of species in the *Heterobasidion* species complex [16,18,19,20]. Studies on the North American species have identified indels in parts of the ITS sequence that could distinguish the species using DNA extracts from pure cultures [16]. Likewise, polymorphisms in alleles of the Elongation Factor alpha and presence/absence of DNA insertions in the ML5-ML6 region of the mitochondrion can be used to differentiate among European species and between European and North American species [18,19]. Other assays can differentiate Eurasian species using taxon-specific primers [21]. These primers are simple to use and have made forest management much easier in the Baltic region, as well as having some forensic applications [22,23].

The objective of this investigation was to develop PCR primers in order to detect and differentiate the North American *Heterobasidion* species collected from various substrates, including infected wood, sporocarps, and cultured isolates. 

## 2. Results

**Figure 1:**
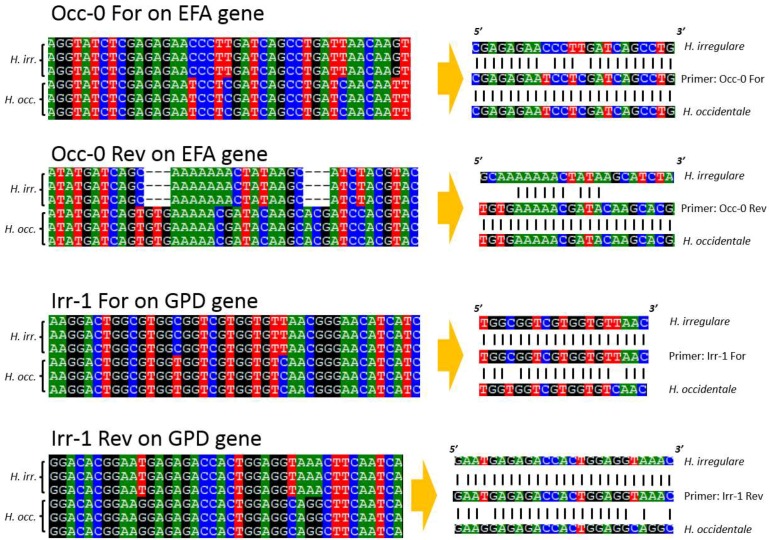
Consensus sequence alignment of *H. irregulare* and *H. occidentale* with the *occidentale*-specific (Occ-0 For and Occ-0 Rev) and the *irregulare*-specific (Irr-1 For and Irr-1 Rev) primers.

**Figure 2:**
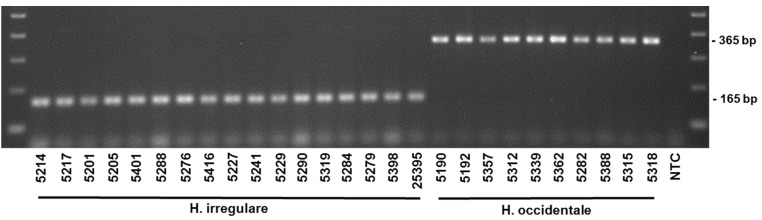
Differentiation of *Heterobasidion* species from pure cultures and herbarium collection with isolates collected across their range in North America. (Note: Isolate 25395 is a herbarium collection- DAVFP25395).

**Figure 3:**
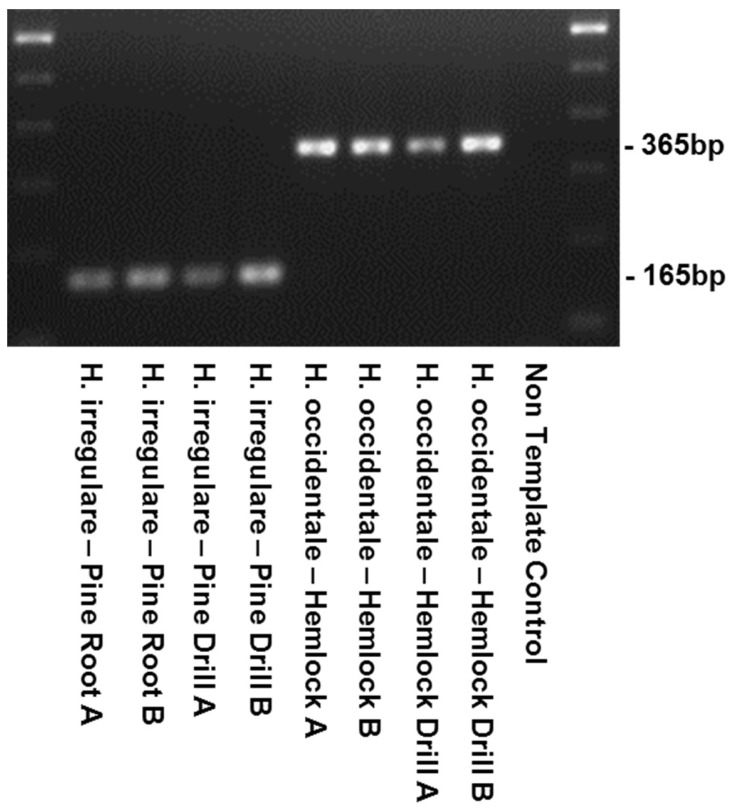
Verification of primers from the infected root and heartwood tissue of diseased trees infected with *Heterobasidion irregulare* or *H. occidentale*.

Alignments of EFA and GPD DNA sequences of 18 isolates of *H. irregulare* and 10 isolates of *H. occidentale* were analyzed for the presence of interspecific polymorphisms. Variable blocks between the two species were identified for both genes and are shown, together with primer locations and sequences, in Figure 1. The two differentiation primer sets were tested on several North American pure cultures (Table 1), herbarium samples and on infected wood samples (Figure 2 and Figure 3). The Irr-1 For and Irr-1 Rev primer set was highly specific and only amplified a 165 base pair (bp) amplicon from *H. irregulare*. DNA from all *H. irregulare* isolates/samples tested amplified successfully. The Occ-0 For and Occ-0 Rev primer set produced a 365 bp amplicon for the *H. occidentale* samples tested, and never amplified *H. irregulare* samples. There were no instances in which both amplicons were present, nor were amplicons of unexpected length ever produced. The primers were tested with a serial dilution of template DNA to determine the limit of detection of the primers. Using conventional PCR, the limit of detection was 20 pg/μL. In general, most PCR amplifications were carried with a DNA concentration of 20 ng/μL.

**Figure 4:**
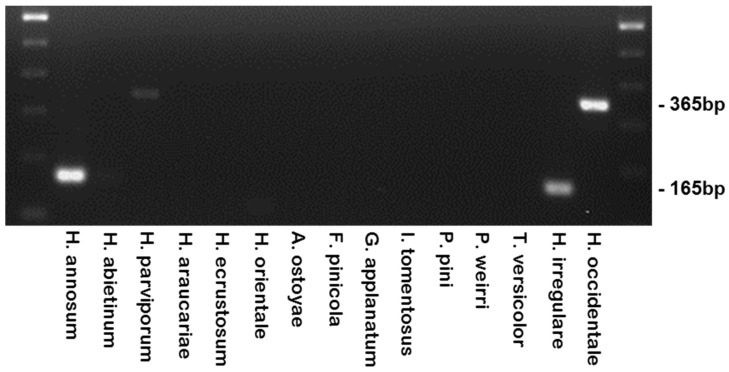
The specificity of the differentiation primers using closely related species from around the world, including other common North American heartwood and root rot fungi.

The primer sets were tested against other species, including the Eurasian species: *H. annosum*, *H. abietinum*, and *H. parviporum* (Figure 4, Table 2). The Irr-1 For and Irr-1 Rev primers did amplify a 165 bp band when tested on *H. annosum* isolates, and the Occ-0 For and Occ-0 Rev primers amplified the expected 365 bp amplicon from *H. parviporum* and *H. abietinum* isolates, although when amplicons were visualized through agarose gel electrophoresis, the bands from *H. abietinum* isolates were significantly “weaker”. *Heterobasidion ecrustosum* and *H. orientale* of the *H. insulare* complex collected in Japan, as well as *H. araucariae* from New Zealand were tested and failed to produce a PCR product (Figure 4, Table 2).

**Figure 5:**
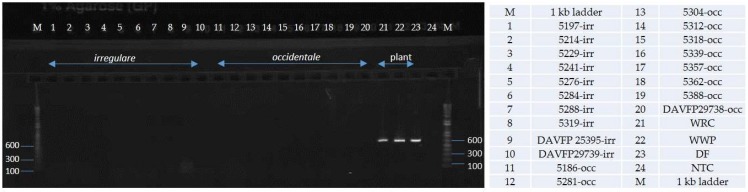
Validation of specificity of universal plant primers PC-UPC-7 For and PC-UPC-7 Rev. irr = *Heterobasidion irregulare* isolates; occ = *Heterobasidion occidentale* isolates; WRC = western red cedar; WWP = western white pine; DF = Douglas fir; NTC = no template control.

**Figure 6:**
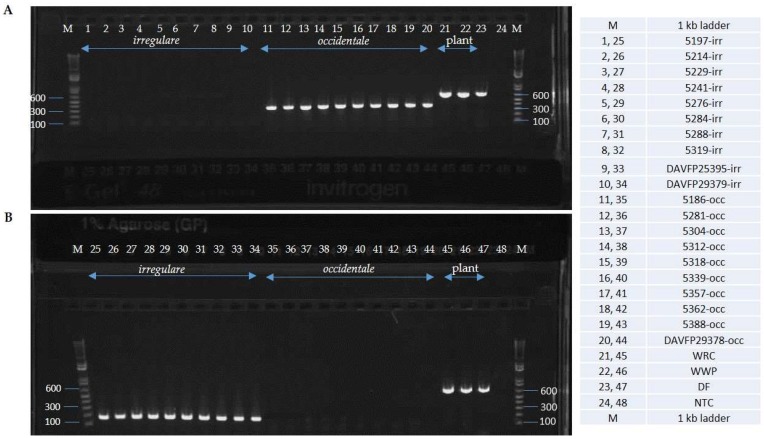
Duplex-PCR using universal plant primers PUC-UPC7 For and PUC-UPC7 Rev and occidentale-specific primers Occ-0 For and Occ-0 Rev (**A**) and universal plant primer PUC-UPC7 For and PUC-UPC7 Rev and irregulare-specific primers Irr-1 For and Irr-1 Rev (**B**). irr = *Heterobasidion irregulare* isolates; occ = *Heterobasidion occidentale* isolates; WRC = western red cedar; WWP = western white pine; DF = Douglas fir; NTC = no template control.

**Figure 7:**
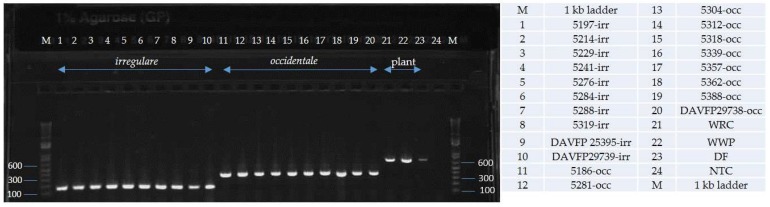
Triplex PCR using universal plant primers PUC-UPC7 For and PUC-UC7 Rev, occidentale-specific primers Occ-0 For and Occ-0 Rev and irregulare-specific primers Irr-1 For and Irr-1 Rev. irr = *Heterobasidion irregulare* isolates; occ = *Heterobasidion occidentale* isolates; WRC = western red cedar; WWP = western white pine; DF = Douglas fir; NTC = no template control.

In addition to DNA extracted from mycelia of pure *Heterobasidion* cultures, the diagnostic primers were successful in amplifying DNA from basidiocarp herbarium samples that were collected in British Columbia, Canada, more than 20 years ago (Table 1, Figure 2, Figure 5, Figure 6 and Figure 7) on *Abies* and *Pinus strobus* and *P. ponderosa*. Basidiocarps collected from *Abies in* 2013 (DAVFP29738; Table 1) only amplified using the *occidentale*-specific primers (Occ-0 For and Occ-0 Rev), while all three basidiocarps collected from *Pinus* in 2013 and 1997 (DAVFP29739, DAVFP29740 and DAVFP25395, respectively) only amplified using the *irregulare*-specific primers (Irr-1 For and Irr-1 Rev) (Figure 6 and Figure 7; data not shown for DAVFP29740). This is the first report of *H. irregulare* in British Columbia.

The specificity of the primers was tested against other forest rot species commonly found in North America. The root rot fungi *Armillaria ostoyae,* the tomentosus root rot fungus (*Onnia (Inonotus) tomentosa*)*,* and the laminated root rot fungus (*Phellinus weirii*) were not amplified by either set of primers (Figure 4). The red ring rot fungus (*Porodaedalae pini*), the white mottled rot fungus (*Ganoderma applanatum*), *Trametes versicolor*, as well as the polypore brown crumbly rot fungus (*Fomitopsis pinicola*) were not amplified by either primer set. All these root rot fungi were collected from coastal British Columbia forests, except for *Onnia tomentosa* was collected from northern British Columbia forests near the Prince George region. Thus, there was no cross-reactivity with any of the non-*Heterobasidion* species tested (Figure 4), however DNA was available and amplifiable from all samples tested as demonstrated by the successful amplification of the internal transcribed spacer (ITS) of all control species using primers ITS-1Fand ITS4 (Table 3) and PCR conditions described by [24,25].

**Figure 8:**
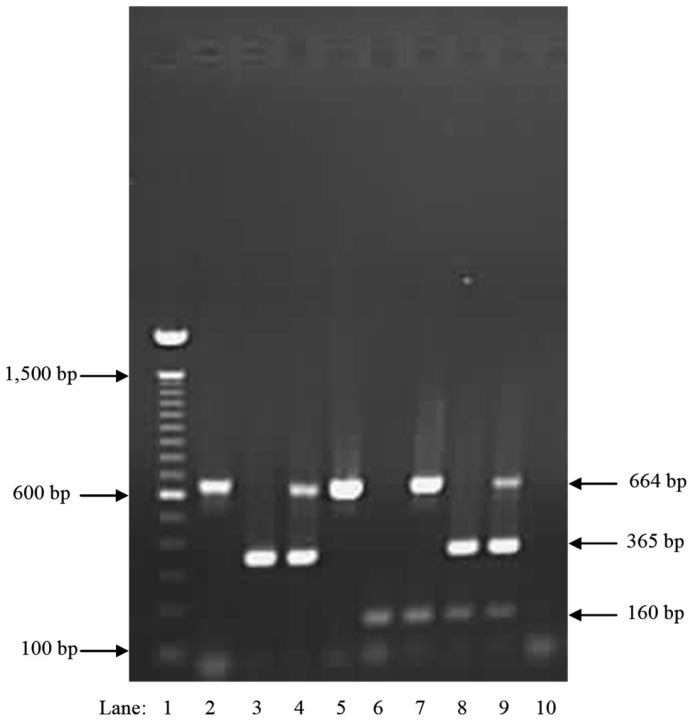
Agarose gel electrophoresis. DNA extracted from colonized wood tissue by North American *Heterobasidion* species. Lane 1—100 bp ladder; lane 2—red alder (band 664 bp); lane 3—*H. occidentale* (band 365 bp); lane 4—red alder and *H. occidentale* (bands 664 bp and 365 bp); lane 5—red pine (band 664 bp); lane 6—*H. irregulare* (band 160 bp); lane 7—red pine and *H. irregulare* (bands 664 bp and 160 bp); lane 8—*H. occidentale* and *H. irregulare* (bands 365 bp and 160 bp); lane 9—red alder, *H. occidentale* and *H. irregulare* (bands 664 bp, 365 bp and 160 bp); lane 10—negative control, no DNA (no bands).

Validation of the primers using an additional internal universal plant primer PUC-UPC7 For and PUC-UPC7 Rev (Table 3) confirmed that DNA of both fungi and plant were amplifiable and showed no cross-amplification when done in single, duplex or triplex PCR (Figure 5, Figure 6 and Figure 7). Finally, when combining red alder or red pine wood with DNA of the two *Heterobasidion* species in the same sample, the three expected specific amplicons were amplified by running in multiplex the three primer sets Irr-1, Occ-0 and PC-UPC7 (Figure 8).

## 3. Discussion

The identification of North American *Heterobasidion* to the species level using the simple PCR protocol described in this paper has allowed us to diagnose both North American species directly from infected woody tissue, saving time and costs compared to culture-based methods. When performed in duplex or triplex, these two species-specific primers do not cross-amplify plant DNA and can be reliably used on environmental samples. Further, the small product size also allowed for the testing of preserved herbarium collections, where DNA integrity is low and conventional PCR methods are troublesome.

The two primer sets differentiate the two North American species, but they do cross-react with all three Eurasian species of the *Heterobasidion annosum* complex, thus, unfortunately, these primers would not be able to differentiate *H. annosum* s.s. from *H. irregulare*, nor *H. parviporum* from *H. abietinum* in those parts of Europe where multiple species exist.

Using this novel method, we have identified *H.* irregulare for the first time on ponderosa pine and eastern white pine in the Okanagan Valley of British Columbia. The first detection of *H. irregulare* on British Columbia’s pine species is specifically significant as *H. irregulare* is considered the most aggressive pathogen in the entire species complex [10]. It is unknown whether: (1) BC isolates may represent a historical Northern boundary of the Western US population, and may have existed there a significant time period without our knowledge; (2) BC isolates represent may be a recent natural Northward expansion of the Western US population, maybe associated with climate change; or (3) BC isolates may be the result of human transport, likely via the planting of infected saplings, and may, thus, represent a geographically and genetically disjunct population from the Western US one.

Although we acknowledge that the significance and rate of occurrence of *H. irregulare* on ponderosa pine and eastern white pine is unknown and needs further investigation, forest management in this area may have to adjust to the presence of this pathogen as done in the USA and Eastern Canada. Additionally, pine species are important export species for Canada. The potential presence of *H. irregulare* in British Columbia pines is of high phytosanitary concern now that *H. irregulare* is on the list of regulated organisms by the European and Mediterranean Plan Protection Organization (EPPO) A2 list of pests recommended for regulation as quarantine pests in September 2015 (http://www.eppo.int/QUARANTINE/Pest_Risk_Analysis/PRA_intro.htm).

The primers and PCR assays described in this study could be used by the Canadian Food Inspection Agency (CFIA) to certify timber and plants as *Heterobasidion*-free, thus, facilitating Canadian export of pines, as EPPO national members will start, including *H. irregulare*, as a fully regulated pathogen in their trade policies. This will also assist the provincial forestry agencies to take suitable control measures to prevent threats to timber production and sustainability of forest production. Furthermore, the assay here described is a cost-effective, fast, and reliable method for detecting and differentiating the two North American *Heterobasidion* species. Thanks to this assay, it was also possible to identify *H. irregulare* for the first time in British Columbia, Canada. There are multiple advantages provided by this new diagnostic method: (1) Current distribution of *Heterobasidion* in North America can be mapped to the species level; (2) wood for export can be tested specifically for the presence of *Heterobasidion*; and (3) species-level diagnosis of *Heterobasidion* may help formulate better disease management strategies.

## 4. Materials and Methods 

### 4.1. Study Sites and Isolates

A representative selection of North American *Heterobasidion* isolates in pure culture was used in this study (Table 1). Isolates were chosen to best represent diverse regions and host species within Canada and the United States. Herbarium samples and infected wood samples from British Columbia (Canada) were also included in the study. DNA from herbarium specimens was obtained by excising 50–100 mg from dry basidiocarps with a sterile blade and carefully excluding tissue from the outside surface of the sample to minimize contamination. Samples of wood infected by *H. occidentale* were collected from wind-thrown or from standing western hemlocks in Mt. Doug and Sandcut Beach municipal forests (Victoria, British Columbia). A hatchet was used to remove the bark, and diseased wood was collected for DNA extraction. Roots were drilled, and the drill shavings were used for DNA extraction. Samples of *H. irregulare* infected wood were collected from two trees a red pine plantation in Lacrosse County, Wisconsin as follows. Infected wood was collected by drilling 5 cm into infected trees near the root collar and collecting the drill shavings generated for DNA extraction [28,29]. Finally, roots of diseased trees were excavated, and a cross-section from each was collected for DNA extraction.

### 4.2. DNA Extraction

DNA was extracted from isolates grown on 2% Malt Extract (Difco) and grown at room temperature for 5–7 days. Approximately 50–100 mg of mycelia was harvested, centrifuged to remove excess liquid and transferred to a sterile 2–mL lysing matrix A tube (MP Biomedical; Solon, OH). The tissues were frozen in liquid nitrogen and homogenized for 10 seconds at 4 m/s using a FastPrep-24 5 G benchtop homogenizer (MP Biomedicals; Solon, OH). Wood samples were similarly treated, frozen in liquid nitrogen, but homogenized twice to pulverize the tissue completely. Conversely, for herbarium samples, homogenization speed and time was reduced to avoid shearing of DNA. DNA of samples of *H. irregulare* infected red pine tissues from Wisconsin was extracted at the Wisconsin Department of Natural Resources in Fitchburg, WI. DNA was extracted using a modified. CTAB extraction protocol with choloroform and ethanol washes [30].

### 4.3. PCR, Sequencing and Primer Design

Two loci, namely the elongation factor 1 alpha and the glyceraldehyde 3-phosphate dehydrogenase, were sequenced from a selection of North American *Heterobasidion* cultures (Table 1). Sanger sequencing was done directly from PCR products using big dye terminators on the ABI 5730xl Data Analyzer at Centre hospitalier de l’Université Laval, Quebec, Canada. Sequences were aligned with the ClustalW extension of BioEdit and edited manually. All sequences used in this study are available in GenBank, while alignments are available from TreeBase (submission ID 17241; www.treebase.org). Allelic blocks that were highly divergent between the two species were identified in the final alignments and selected for primer design. Two sets of candidate species-specific primers, namely, Irr-1 For and Irr-1 Rev and Occ-0 For and Occ-0 Rev (Table 3) were designed manually from these regions focusing on indels and high variation at the 3’ end of the primer. To ascertain that DNA extractions from both pure cultures and environmental samples were successful, a pair of universal plant-specific primers (Table 3) derived from the chloroplast genome (http://bfw.ac.at/200/2043.html) was used as an internal control during the validation step.

### 4.4. Primer Testing and Validation of the Assay

The two differentiation primer sets (Table 3), namely Irr-1 For and Irr-1 Rev and Occ-0 For and Occ-0 Rev, were tested for their specificity using DNA extracted from pure cultures and herbarium samples of North American *Heterobasidion* species (Table 1). Amplification was performed using differentiation primer sets Irr-1 For and Irr-1 Rev and Occ-0 For and Occ-0 Rev in duplex. An aliquot of 1.0 µL of diluted genomic DNA was included in each 25.00 µL PCR reaction (1X PCR reaction buffer, 1.5 mM MgCl_2_, 1U of Platinum *Taq* Polymerase [Invitrogen, Carlsbad, CA], 0.25 µM of each primer (Integrated DNA Technologies), 0.3 mM dNTPs [Invitrogen]). A minimum of one negative water control was included with each PCR run. Amplifications were carried out in a Veriti 96-well thermal cycler (Applied Biosystems, Carlsbad, CA)) under the following conditions: Initial denaturation at 94.0 °C for 5 min; then 35 cycles of denaturation at 94.0 °C for 30 s, annealing for 40 s (annealing temperatures in Table 3), extension at 72.0 °C for 55 s; and a final extension at 72.0 °C for 5 min. The differentiation primer sets Irr-1 For and Irr-1 Rev and Occ-0 For and Occ-0 Rev were always used together in the same reaction, each at 0.25 µM. PCR products were visualized using electrophoresis with 1.5% agarose gels stained with ethidium bromide.

Further validation of the differentiation primers sets Irr-1 For, Irr-1 Rev, Occ-0 For and Occ-0 Rev were done on DNA extracted from pine and hemlock wood infected by *H. irregulare* and *H. occidentale,* respectively; on DNA from pure cultures of closely-related *Heterobasidion* species; as well as on DNAs of common North American heartwood and root rot fungi. Amplification was conducted using identical PCR conditions as stated above using the primer sets Irr-1 For, Irr-1 Rev, Occ-0 For and Occ-0 Rev, in duplex PCR reaction.

A third validation assay using the two *Heterobasidion* differentiation primers and the internal plant-specific primer (Table 3) was performed to confirm that DNA extractions were successful, and the differentiation primers did not cross-amplify plant DNA. We used DNA from pure cultures and herbarium samples of North American *Heterobasidion* (Table 1), as well as DNA from representative conifer species. The PCR was run using the three sets of primers in single-, duplex and triplex.

A fourth validation assay, using three primer tools, was also tested on samples containing DNA from *H. occidentale*, *H. irregulare*, and wood shavings from red alder (*Alnus rubra*) and red pine (*Pinus resinosa*) in all possible combinations.

## Figures and Tables

**Table 1:** North American *Heterobasidion* culture isolates and herbarium samples used in this study.

Species	Isolate or Herbarium Collection Number	Geographic Origin	Host	Year Collected	Source/Collector	ITS	EFA	GPD
*irregulare*	PFC5201	Limerick, ON, Canada	*Pinus resinosa*	2003	M. Dumas	KP863563	KP863594	KP863623
*irregulare*	PFC5205	York, ON, Canada	*P. resinosa*	2005	M. Dumas	KP863564	KP863596	KP863625
*irregulare*	PFC5214	St. Philippe, QC, Canada	*P. resinosa*	2002	G. Laflamme	KP863565	KP863597	KP863626
*irregulare*	PFC5217	Lac La Blanche, QC, Canada	*P. resinosa*	2002	G. Laflamme	KP863566	KP863598	KP863627
*irregulare*	PFC5227	Iowa Co, WI, USA	*P. resinosa* or *P. strobus*	1994	G. Stanosz	KP863567	KP863599	KP863629
*irregulare*	PFC5229	Union Co, IL, USA	*P. echinata*	1994	G. Stanosz	KP863568	KP863600	KP863630
*irregulare*	PFC5241	Portage Co, WI, USA	*Abies balsamea*	2010	G. Stanosz	KP863571	KP863602	KP863634
*irregulare*	PFC5276	S. Pines, NC, USA	*P. taeda*	1967	J.S. Boyle	KP863572	KP863603	KP863635
*irregulare*	PFC5279	Lassen Natl. Forest, CA, USA	*P. ponderosa*	1981	J. Worrall	KP863573	KP863604	KP863636
*irregulare*	PFC5284	San Bernardino Mtns., CA, USA	*P. jeffreyii*	1975	J. Worrall	KP863574	KP863605	KP863639
*irregulare*	PFC5288	Durham College Woods, NH, USA	*Juniperus virginiana*	1987	T. Harrington	KP863575	KP863606	KP863641
*irregulare*	PFC5290	Nebraska, USA	*P. ponderosa*		J. Blodgett	KP863576	KP863607	KP863642
*irregulare*	PFC5319	Montrose Co, CO, USA	*P. ponderosa*	2006	J. Worrall	KP863581	KP863611	KP863647
*irregulare*	PFC5401	St. Williams, ON, Canada	*Thuja plicata*		D.C. Constable	KP863587	KP863617	KP863653
*irregulare*	PFC5416	Warren Co, GA, USA	*Spore trap*	2007	M. Cram	KP863589	KP863619	KP863655
*irregulare*	DAVFP25395	Okanagan Falls, BC, Canada	*P. ponderosa*	1997	J. Hodges	KP863588	KP863618	KP863654
*irregulare*	DAVFP29739	Summarland, BC, Canada	*P. strobus*	2013	J.H. Ginns			
*irregulare*	DAVFP29740	Summerland, BC, Canada	*P. strobus*	2013	J.H. Ginns			
*occidentale*	PFC5190	Ladysmith, BC, Canada	*Tsuga heterophylla*		B. Callan	KP863561	KP863592	KP863621
*occidentale*	PFC5192	Jordan River, BC, Canada	*T. heterophylla*		B. Callan	KP863562	KP863593	KP863622
*occidentale*	PFC5282	Modoc Natl Forest, CA, USA	*A. conc* *olor*	1981	J. Worrall	KP492941.1	KP571672.1	KP863638
*occidentale*	PFC5312	King Co, WA, USA	*T. heterophylla*	2011	R. Edmonds	KP863578	KP863608	KP863644
*occidentale*	PFC5315	Ouray Co, CO, USA	*A. concolor*	2005	J. Worrall	KP863579	KP863609	KP863645
*occidentale*	PFC5318	Mineral CO, CO, USA	*A. concolor*	2005	J. Worrall	KP863580	KP863610	KP863646
*occidentale*	PFC5339	OR, USA	*A. concolor*	2009	E. Goheen	KP863582	KP863612	KP863648
*occidentale*	PFC5357	Clallam Co, WA, USA	*T. heterophylla*	2011	R. Edmonds	KP863583	KP863613	KP863649
*occid* *entale*	PFC5362	Lincoln Co, OR, USA	*T. heterophylla*	2011	M. Elliott	KP863584	KP863614	KP863650
*occidentale*	PFC5388	San Bernardino, CA, USA	*A. concolor*	2011	P. Zambino	KP863585	KP863615	KP863651
*occidentale*	DAVFP29738	Summerland, BC, Canada	*Abies stump*	2013	J.H. Ginns			

Isolate preceded by PFC (Pacific Forestry Centre) are pure culture sample, and DAVFP (Department of Agriculture, Victoria, Forest Pathology) are herbarium samples.

**Table 2:** List of Eurasian isolates used in this study.

Species	Isolate or Herbarium Collection Number	Geographic Origin	Host	Year Collected	Source/Collector	ITS	EFA	GPD
*abietinum*	PFC5247	Poland	*Abies alba*		H. Solheim	KC492895.1	KC571636.1	KP863657
*abietinum*	PFC5249	Austria	*Picea abies*		H. Solheim	KC492896.1	KC571637.1	KP863658
*abietinum*	PFC5373	Greece	*Abies cephallonica*	1993	P. Tsopelas	KC492956.1	KC571687.1	KP863664
*annosum*	PFC5252	Norway	*Pinus sylvestris*	1937	R.H. Roll-Hansen	KC492906.1	KC571646.1	KP863659
*annosum*	PFC5257	Italy	*Pinus pinaster*	2008	A. Biraghi	KC492909.1	KC571649.1	KP863660
*annosum*	PFC5260	Serbia	*Pinus nigra*	2003	D. Dubak	KC492911.1	KC571651.1	KP863661
*araucariae*	PFC5434	New Zealand	*Agathis australis*	1958	J.W. Gilmour	KX130098	KX130101	KX130104
*ecrustosum*	PFC5438	Japan	*Pinus thunbergii*		P.K. Buchanan	KX130099	KX130102	KX130105
*orientale*	PFC5439	Japan	*Tsuga sp.*		P.K. Buchanan	KX130100	KX130103	KX130106
*parviporum*	PFC5262	Norway	*Picea abies*	2004	R. Saursaunet	KC492957.1	KC571688.1	KP863662
*parviporum*	PFC5269	Japan	*Abies mayriana*	1942	S. Kamei	KC492951.1	KC571682.1	KP863663
*parviporum*	PFC5293	Russia	*Abies sibirica*		K. Korhonen	KC492913.1	KC571653.1	KP863665

**Table 3:** Details about the primers used in this study.

Primer	Sequence	Gene	Product (bp)	Tm (°C)	Reference
Irr-1 For	TGGCGGTCGTGGTGTTAAC	GPD	165	64	This study
Irr-1 Rev	GAATGAGAGACCACTGGAGGTAAAC	GPD	165	64	This study
Occ-0 For	CGAGAGAATCCTCGATCAGCCTG	EFA	365	64	This study
Occ-0 Rev	TGTGAAAAACGATACAAGCACG	EFA	365	64	This study
ITS1-F	CTTGGTCATTTAGAGGAAGTAA	ITS	486/515	55	[24]
ITS4	TCCTCCGCTTATTGATATGC	ITS	486/515	55	[25]
EFA For	TCAACGTGGTCGGTGAGCAGGTA	EFA	447-54	66	[26]
EFA Rev	AAGTCACGATGTCCAGGAGCATC	EFA	447-54	66	[26]
GPD-Seq For	CAGAGCCTCTGCCCACTTGAAGG	GPD	666/754	59	This study
GPD-Seq Rev	GCCGGGTGGCCGACAAAGTC	GPD	666	59	This study
PC-UPC7 For	GGATTRCGTATGGGMAATATTGAAAC	CHLOROPLAST	664	64	[27]
PC-UPC7 Rev	CCCCTTGGACTRCTACGAAAAACACC	CHLOROPLAST	664	64	[27]
